# Genome-wide expression analysis of *LBD* genes in tomato (*Solanum lycopersicum* L.) under different light conditions

**DOI:** 10.1080/15592324.2023.2290414

**Published:** 2023-12-07

**Authors:** Limei Dong, Hakim Manghwar

**Affiliations:** a Guangdong Eco-engineering Polytechnic, Guangzhou, Guangdong, P.R. China; b Lushan Botanical Garden, Jiangxi Province and Chinese Academy of Sciences, Jiujiang, Jiangxi, P.R. China

**Keywords:** Tomato plants, LBD gene family, LEDs, Expression analysis

## Abstract

Lateral organ boundaries (LOB) domain (*LBD*) genes, a gene family that encodes the transcription factors (TFs) of plants, plays crucial functions in the development and growth of plants. Currently, genome-wide studies of the *LBD* family are still limited to tomato (*Solanum lycopersicum* L.), which is considered an important economic crop. In this study, we performed a genome-wide analysis of *LBD* in tomato. In total, 56 *LBDs* were found in the tomato genome. Protein alignment and phylogenetic classification showed that *LBDs* were conserved with other species. Since light emitting diodes (LEDs) light have promising applications for tomato growth. To better understand the potential function of *LBDs* in response to LED light in tomato, we conducted a genome-wide expression analysis of *LBD* genes under different light conditions. As expected, different LED lights affected the tomato growth (e.g. hypocotyl length). RNA-seq data showed that eight *LBDs* in tomato seedlings were differentially expressed under different light treatments, including white, blue, red, and far-red light, compared to the dark-grown condition. It indicates that these *LBDs* might regulate plant development in different LED light conditions. Interestingly, two *LBD* genes (*SlLBD1* and *SlLBD2*) were found to be differentially expressed in four distinct lights, which might be involved in regulating the plant architecture via a complicated TF network, which can be taken into consideration in further investigation.

## Introduction

Tomato (*Solanum lycopersicum* L.) is a highly popular vegetable species across the globe, which has been grown in greenhouses that make its production possible throughout the year.^1^ To achieve fruits with good taste and high yields, it is recommended to maintain a daily light integral (DLI) of 20–30 mol m^−2^ day.^2^ Tomato is usually grown off-season in greenhouses in regions like China, northern Europe, and the USA that experience low solar radiation and shorter days However, off-season tomato fruits grown in the greenhouse are found to have less flavor and taste than those in-season produced in the field.^3^

Light has a crucial role in the survival of plants, and it helps them in building biomass and synthesizing organic compounds.^1^ It also determines morphogenesis and plant growth, such as circadian rhythms through photoreceptors and hormones.^4,5^ Changes in the spectral composition of light significantly affect various biological activities, including photosynthesis to secondary metabolism.^6,7^ Light-emitting diodes (LEDs) are being increasingly used in greenhouses to enhance the quality and production of plants. LEDs provide the ability to accurately control the spectral composition of supplemental light.^8^ Plants can perceive the entire solar light spectrum, which ranges from UV to far-red, and this has been extensively studied for its impact on plant growth and development.^9^

Transcription factors (TFs) play an essential role in regulating various developmental processes in plants, including signal transduction, cell morphogenesis, and response to environmental stresses by modulating gene expression.^10^ The lateral organ boundaries (LOB) domain (LBD) proteins possess a distinctive N-terminal LBD.^11,12^ Currently, LBD has been found only in the plant genome, indicating its sole involvement in regulating developmental processes in plants.^12,13^

In order to understand the potential role of LED TF in shaping the process of developmental progress of tomatoes in response to LED lights. In this study, we performed a genome-wide expression analysis of *LBD* genes in the tomato genome and constructed a potential regulatory network of *LBD* genes. Eight *LBDs* were differentially expressed in tomato under the condition of different lights, such as white, blue, red, and far-red light as compared to the dark-grown condition, indicating the role of *LBD* in plant development. Two *LBD* genes, namely *SlLBD1* and *SlLBD2* were observed to show differential expression in four different lights, which might regulate the plant architecture through a complex TF network that can be considered for further investigation.

## Materials and methods

### LBD prediction, alignment, and phylogenetic tree construction

The general feature format (GFF), coding gene sequences, genome assembly, etc., of tomato were obtained from the Sol Genomics Network Database (https://solgenomics.net/organism/Solanum_lycopersicum/genome). The identification of TFs, such as *LBD* was conducted through the utilization of the iTAK program.^14^ TBtools (https://github.com/CJ-Chen/TBtools) was used to display the *LBD* gene structure. For sequence alignment, the CLC Sequence Viewer software (https://www.qiagenbioinformatics.com/products/clc-sequence-viewer/) was utilized. At the same time, MEGA7 was used to construct the phylogenetic tree,^15^ and the iTOL tool (http://itol.embl.de) was utilized to process the tree.

### Gene structure and conserved motif analysis

The distribution of exons and introns of each tomato *LBD* gene was examined by comparing their predicted coding sequences with their corresponding genomic sequences by using the Gene Structure Display Server 2.0 (http://gsds.cbi.pku.edu.cn/).^16^ The online software MEME (http://meme-suite.org/tools/meme) with default parameters was utilized to investigate the conserved motifs of tomato LBD protein sequences.^17^

### Transcriptome library preparation

Seedlings subjected to various light treatments were separately sampled and mixed for the extraction of a total RNA by utilizing the RNAprep Pure Plant Kit (Tiangen, China), following instructions provided by the manufacturer. To pull down the mRNA, the magnetic Dynabeads Oligo (dT)_25_ (Invitrogen, USA) was used with 5 μg total RNA. The VAHTS Universal DNA Library Prep for Illumina V2 Kit (Vazyme, China) was utilized to prepare the libraries for transcriptome and sequenced at the Illumina NovoSeq 6000 platform in paired-end mode (PE150).

### RNA-seq data analysis and network construction

Raw reads were removed from the sequencing adaptors and filtered out the reads with low quality by using the Trim_galore software (https://github.com/FelixKrueger/TrimGalore) with the default parameter. Then, the HISAT2 (http://ccb.jhu.edu/software/hisat2) was used to map the filtered reads to the tomato reference genome ITAG4.0 (https://solgenomics.net/organism/Solanum_lycopersicum/genome). The expression level was calculated using the Fragments Per Kilobase of exon model per Million mapped fragments (FPKM) via cuffnorm (http://cole-trapnell-lab.github.io/cufflinks/cuffnorm/). The genes with log2 (Fold Change) greater than 1 and FDR less than 0.05 were defined as differentially expressed genes (DEGs) and calculated by utilizing the cuffdiff tool (http://cole-trapnell-lab.github.io/cufflinks/cuffdiff/). The heatmaps were generated by using the Pheatmap package in R language (https://cran.rproject.org/web/packages/pheatmap).

To construct a co-expression network between *LBD* and other TFs, genes with an FPKM greater than 1 in any of the samples were selected for calculating the Pearson correlation coefficient (PCC). Only absolute PCC values greater than 0.8 were considered as a potential interaction. The Cytoscape software (http://www.cytoscape.org) was used to visualize the potential network.

### Promoter motif analysis

The position weight matrix (PWM) for the known LBD motif was downloaded from the Plant Cistrome Database (http://neomorph.salk.edu/PlantCistromeDB). Motif scanning was performed using the FIMO program from the MEME suite software package (https://meme-suite.org/meme/).

### Morphological analysis

The wild-type (WT) tomato *cv Ailsa Craig* was used in this study. To measure the hypocotyls’ length of tomato seedlings in different treatments, 7-day-old tomato seedlings were grown in different LEDs light (white, blue, red, and far-red) conditions or complete dark conditions. Images were captured by utilizing a stereomicroscope (LEICA S9D), and ImageJ software was used to measure the hypocotyls’ length.

### Data availability

The raw data obtained from RNA-seq experiments were submitted to the Genome Sequence Archive Database (http://gsa.big.ac.cn) with the assigned BioProject Number PRJCA000689.

## Results and Discussion

### Prediction of LBD genes in the tomato genome

**Figure 1.**
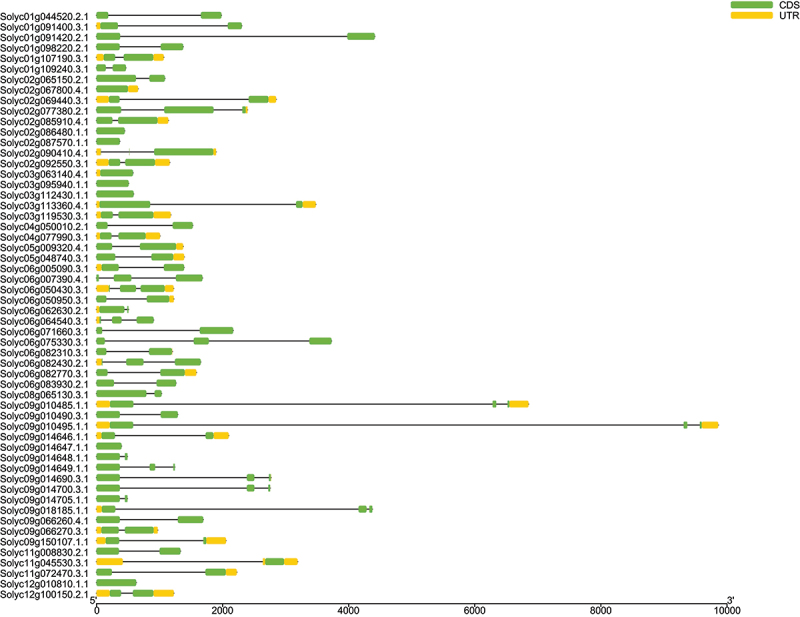
Gene structure of *LBD* genes in the tomato genome.

To identify the *LBD* genes present in the tomato genome, iTAK program^14^ was employed using the deduced protein sequences from the *Solanum lycopersicum* protein sequence (https://solgenomics.net/organism/Solanum_lycopersicum/genome). Our analysis revealed a total of 56 *LBD* genes, which were distributed across 12 chromosomes (Figure 1, Table S1 and S2).

The yellow color indicates gene coding sequences (CDS). The green color indicates untranslated regions (UTR). The scale bar indicates the gene length (bp).

Analyses of exon/intron organization and conserved motifs were conducted in order to understand the structural diversity of *LBD* genes. Gene structure analysis revealed that out of 56 *LBD* genes, 14 were found to lack introns, while the remaining genes exhibited the presence of at least one intron. The exon-intron structure was found to be identical among most of the *LBD* members in the same subfamily (Figure 1, Table S3). Interestingly, the majority of numbers in group I contained only one exon (Figure 1, Table S3), possibly suggesting that they are a specific class of *LBD* of tomato.

**Figure 2.**
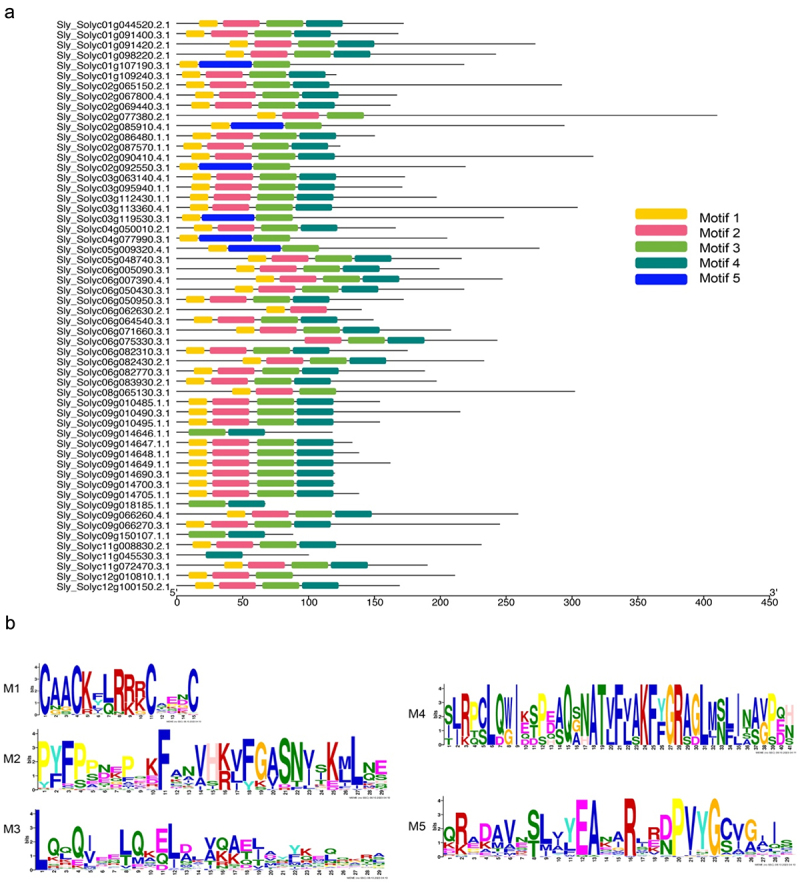
Conserved motifs of LBD proteins in accordance with the phylogenetic relationship.

Additionally, the potential conserved motifs of LBD proteins were further detected by utilizing the MEME program. The MEME program was also used to analyze the putative motifs. Consequently, we identified 5 divergent motifs (M1-M5) in LBD (Figure 2a, b). M1-M4 were the most conserved domain, also known as C block, GAS Block, L-rich Block, and C Block, respectively, existed in most plant species, including tomato, *Brassica Napus*, etc.^18^ M5 existed in some *LBD* genes in tomato, such as *Solyc01g107190.3* and *Solyc02g092550.3*, which might have specific functions that need further investigation (Figure 2).

The conserved motifs in the LBD proteins were identified by MEME. Grey lines represent the non-conserved sequences, and each motif is indicated by a colored box numbered at the bottom. The length of motifs in each protein was displayed proportionally.

### Multiple sequence alignment and phylogenetic analysis

**Figure 3.**
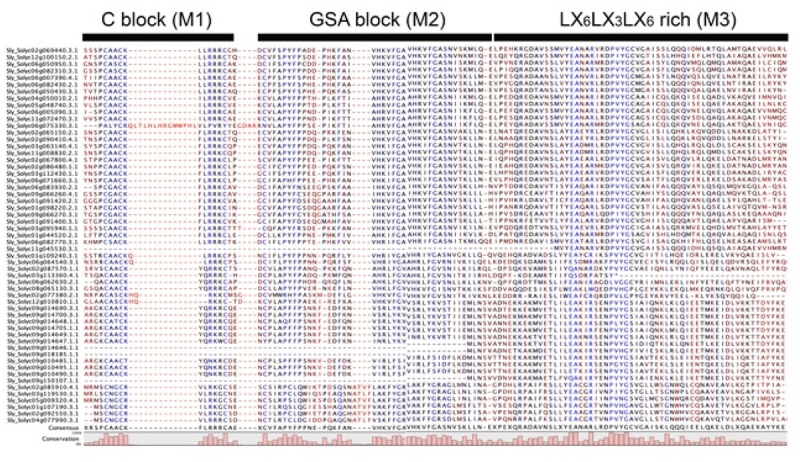
The conserved domains of the *LBD* gene family.

As mentioned above, several conserved motifs have been identified via the MEME program, which were reported previously. To further determine the phylogenetic relationship between these LBD proteins. We first performed the multiple sequence alignment using these LBD proteins. As expected, the C block (CX2CX6CX3C), GAS block that includes glycine (G), alanine (A), and serine (S) in consecutive order, and a leucine zipper-like motif (LX6LX3LX6L, L rich block) have been found after alignment^19^ (Figure 3), which consisted with the M1, M2, and M3 motifs (Figure 2).

The deduced amino acid sequences of LBD protein were aligned, and conserved domains were identified. The C block (M1), GSA block (M2), and L-rich block (M3) were shown as an example here.

**Figure 4.**
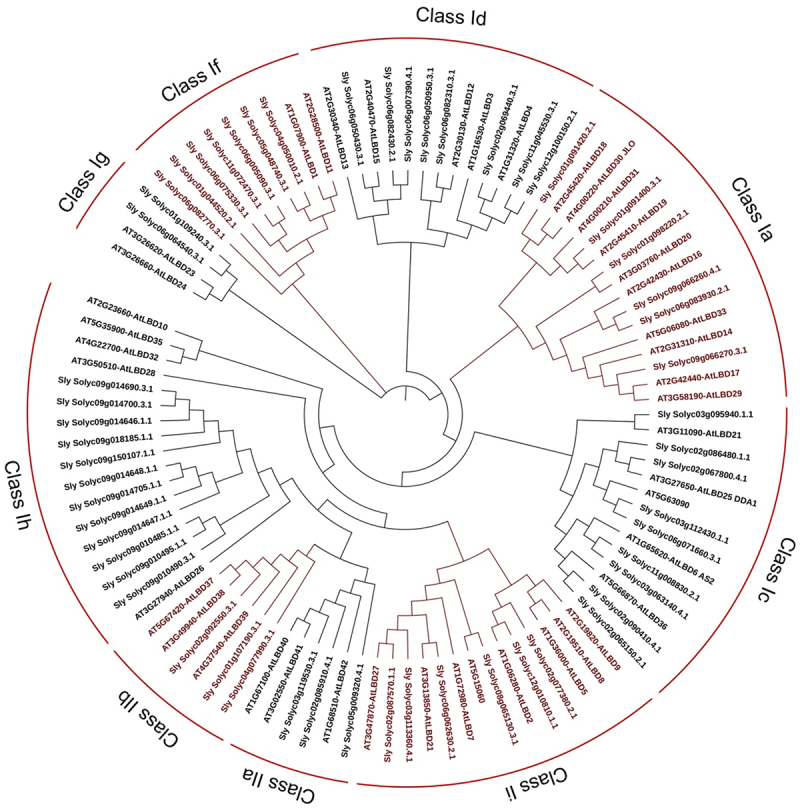
Phylogenetic analysis of tomato and Arabidopsis *LBD* genes.

Furthermore, phylogenetic analysis based on the alignment results reveals that *LBDs* can be divided into two main classes, namely Class I and Class II. Class I can be subdivided into seven subclasses (a, c, d, f, g, h, and i), while Class II comprises two subclasses (a and b), which is consistent with the kinship reported previously^20^ (Figure 4).

Two classes were identified: Class I subdivided into seven subclasses (a, c, d, f, g, h, and i) and Class II into two subclasses (a – b).

In the phylogenetic tree, *Solyc11g0088320* and *Solyc03g063140* were in the same clade as *AtLBD6 (AS2)* in Class Ii, while *Solyc01g091420* formed a clade with *AtLBD30* (JLO) in Class 1a (Figure 4). *AtLBD6* (*AtAS2*) is a crucial member of the LBD gene family, which can form complexes with various proteins for regulating the different aspects of plant growth and development.^21,22^ AS2 interacts with *AtLBD30*/JAGGED LATERAL ORGANS (JLO) in order to regulate the expressions of various *PIN-FORMED (PIN)* genes that encode Aux efflux facilitators.^23^ AS2, *AtLBD36*/AS1, and JLO have the ability to form a trimeric protein complex that plays a role in organ boundary formation by negatively regulating the expression of the *KNOX* gene.^24^ Additionally, the *AtAS2* was found to inhibit cell proliferation in the axial region by regulating the *KNOX* gene, resulting in the symmetrical development of the leaf proximal-distal axis, which forms spreading leaves.^25^ These data together suggested that these tomato *LBD* genes might have similar functions to those homologs in *Arabidopsis*.

### Expression patterns of LBD under different light treatments

As LED light potentially affects plant growth, we wanted to know how *LBD* expression changes under different light treatments and understand the potential regulatory network of *LBD*. In this study, we used a hypocotyl system to measure the RNA level of *LBD* under different light treatments using transcriptomic data. As shown in Figure 4a, the hypocotyls of tomato seedlings have significant differences under different light treatments. To further compare the gene expression in response to different lights, we performed the RNA-seq (Table S4) and compared the red light, white light, blue light, and far-red light samples to those grown under dark conditions.

**Figure 5.**
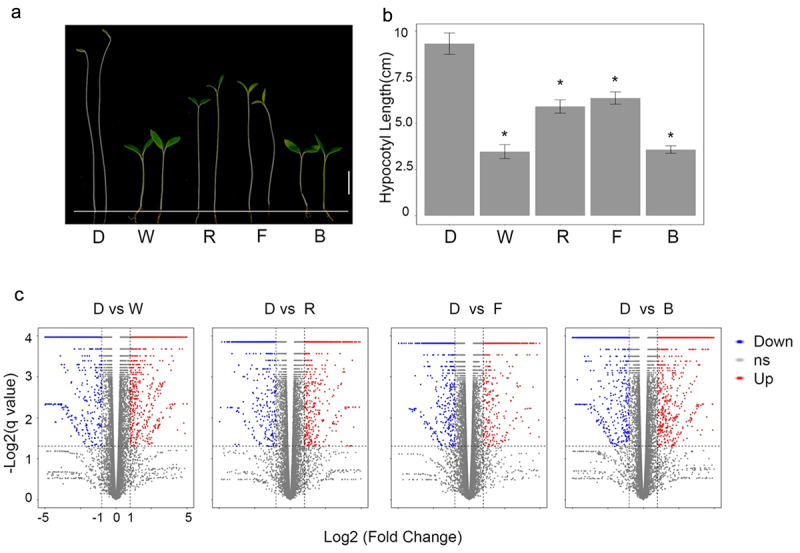
The phenotype of tomato seedlings under different light grown conditions.

**Figure 6.**
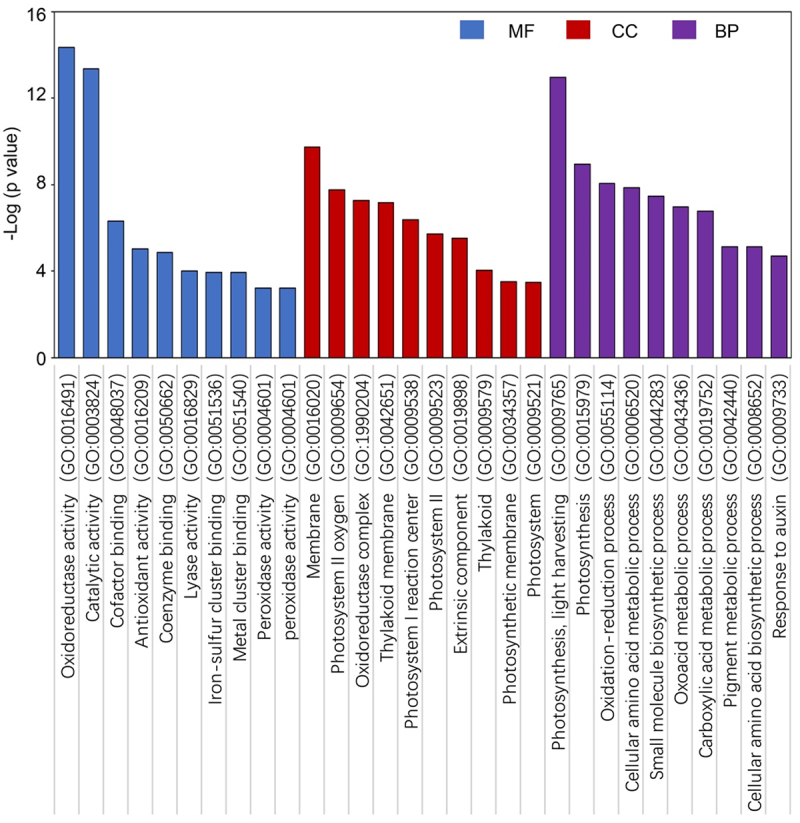
GO enrichment of DEGs in different light treatments compared to dark. The top 10 ranked GO terms categories are shown here. MF: molecular function, CC: cellular component (CC), and BP: biological process.

We found 1974 down-regulated DEGs and 2938 up-regulated DEGs in blue light grown seedlings compared to dark grown seedlings. The 1141 down-regulated and 1609 up-regulated DEGs in far-red grown seedlings were found. The 1134 down-regulated and 1622 up-regulated DEGs in red light grown seedlings and 1965 down-regulated and 3008 up-regulated DEGs in white light grown seedlings were found (Figure 5c, Table S5 and S6). Next, using these DEGs to perform the GO enrichment test, as expected, several light-response-related GO terms are significantly enriched, such as Photosynthesis (GO:00015979), Oxidoreductase activity (GO:16491), and Phytosytem (GO:0009654), indicated the reliability of these RNA-seq data (Figure 6).

(a) The hypocotyl phenotype of tomato seedlings under different light treatments. D, W, R, F, and B indicated the Dark, White light, Red light, Far-red light, and Blue light, respectively, grown tomato seedlings. Bar = 2 cm. (b) The hypocotyl length of tomato seedlings under different light treatments. Asterisk (*) indicates a significant difference compared to dark grown seedlings (*p* <.05, Student’s t-test). The error bar indicated the mean ±SD of 30 plants. (c) The volcano plot shows the DEGs in the RNA-seq data. The cutoff of DEGs is Log2 (Fold Change) >1 and FDR <.05.

**Figure 7.**
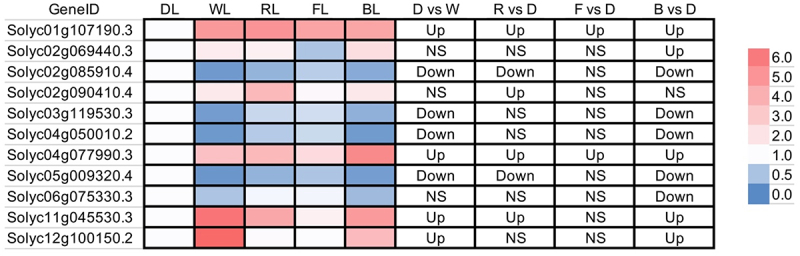
Heatmaps showing the expression level of *LBD* in different light grown conditions.

As for *LBD* genes, we found that 11 out of 56 members were significantly changed during this light treatment (Figure 7). Among them, 6 *LBDs* have upregulation in light (at least in one light) treatment (e.g., *Solyc01g10g7190.3* and *Solyc04g077990*), while 5 *LBDs* (e.g., *Solyc02g085910*) have a high expression trend in dark-grown conditions.

The log2(FPKM values) were used to generate the heatmaps (left panel). The right panel indicated the significant change (Up or down-regulated) of the *LBD* gene in four LED lights compared to dark. NS, not significant; D, Dark; W, White; R, Red; F, Far-red; B, Blue; and L, Light.

### Potential regulatory network of two LBDs

**Figure 8.**
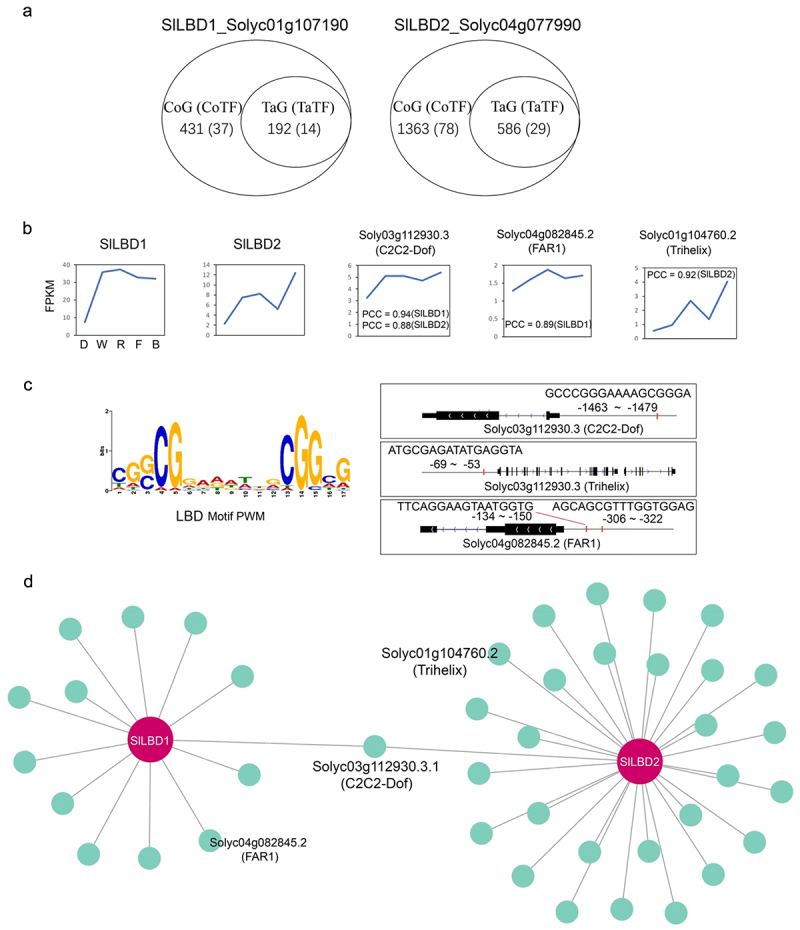
The potential regulatory network of two *LBD* genes.

As mentioned above, *Solyc01g10g7190* and *Solyc04g077990* were upregulated in all light conditions compared to the dark treatment, indicating a potential central role of these *LBDs* in response to light. In this study, we named these *LBD* genes as *SlLBD1* and *SlLBD2*. To identify the potential co-regulating partners of these two *LBDs*, the Pearson correlation coefficient (PCC) was calculated between the expression levels of *LBDs* and those of other genes (Table S7). The *LBD*/genes pair with PCC value over 0.85 were regarded as the potential *LBD* co-regulating partners (Table S7). As shown in Figure 8a, 431 *SlLBD1* and 1363 *SlLBD2* co-expression genes (CoGs) were found. As the *LBD* Motif has been reported, we scanned the promoter of these *LBD* CoGs using the *LBD* Motif Position weight matrix (PWM). Genes promoter that harbored the *LBD* PWM was considered as the *LBD* directly targeted genes (TaG). Among these CoGs, we found 192 *LBD* TaGs, including 14 TFs and 586 TaGs involving 29 TFs (Figure 8a, (Table S7)).

(A) The Pearson Correlation Coefficient (PCC) and motif analysis showing the co-expression genes (CoG) and targeted genes (TaG) of two interesting *LBD* genes, Solyc01g10g7190.3 and Solyc04g077990, which are named as *SlLBD1* and *SlLBD2*. (b) The expression pattern of SlLBD1, SlLBD2, FAR1, Trihelix, and C2C2-dof. (c) The LBD motif position in the promoter of FAR1, Trihelix, and C2C2-dof. The LBD Position weight matrix (PWM) is shown in the left panel, and the motif position and match sequences are shown in the right panel. (d) The potential direct regulatory network of *SlLBD1* and *SlLBD2*. Several TF involved in light response, such as FAR1, Trihelix, and C2C2-dof, were found as potential downstream targets of these two *LBD* genes.

Among the TaGs, several genes, such as the *Solyc01g104760* (Trihelix), *Solyc04g082845* (FAR1), and *Solyc03g112930* (C2C2-Dof) have been reported to play a key role in light responses (Figure 8b, c). The trihelix family is categorized as GT factors because of their binding specificity for GT elements.^26^ Trihelix TFs are involved in a variety of developmental processes, such as light-dependent expression regulation,^27^ roles in various developmental processes like morphogenesis control of various leaves and flower organs,^28^ embryos,^29^ trichomes development,^30^ and responses to biotic and abiotic stresses.^31^

The *far-red-impaired response 1 (FAR1)* transcription family was first identified as a crucial factor for phytochrome A (phyA)-mediated far-red light signaling in Arabidopsis. They play a significant role in regulating plant growth and development.^32^ Studies on gene expression regulation have shown that *FAR1* plays a vital role in light signal transduction and regulates the growth and development of plants, defense, and immunity.^33,34^

Members of the Dof (DNA-binding one zinc finger) is a TF family consisting of proteins with a highly conserved DNA-binding domain known as the Dof domain, containing a C2C2 zinc-finger motif.^35^ Dof proteins play a crucial role in various physiological processes in plants, such as hormone responses, phytochrome signaling, lipid synthesis, seed germination, carbohydrate metabolism, flowering time regulation, seed dormancy, leaf senescence, floral vasculature, and resistance to salt stress and powdery mildew.^36^ Finally, we constructed a potential *LBD1/2* regulatory network regulating the downstream expression in response to different light conditions, which needs further investigation.

## Supplementary Material

Table S1 TF prediction results from iTAK program.xlsx

Table S3 LBD Gene Description.xlsx

Table S6 DEG information.xlsx

Table S4 RNA seq Raw Data Information.xlsx

Table S5 All Gene expression levels in different lights.xlsx

Table S7 LBD Coexpressed and Targeted Genes information.xlsx

Table S2 LBD gene location.xlsx
